# Human Liver Stem Cell-Derived Microvesicles Inhibit Hepatoma Growth in SCID Mice by Delivering Antitumor MicroRNAs

**DOI:** 10.1002/stem.1161

**Published:** 2012-06-26

**Authors:** Valentina Fonsato, Federica Collino, Maria Beatriz Herrera, Claudia Cavallari, Maria Chiara Deregibus, Barbara Cisterna, Stefania Bruno, Renato Romagnoli, Mauro Salizzoni, Ciro Tetta, Giovanni Camussi

**Affiliations:** a Department of Internal Medicine, Research Center for Experimental Medicine (CeRMS) and Center of Molecular BiotechnologyTorino, Italy; b Liver Transplantation Center, University of TorinoTorino, Italy; c Fresenius Medical CareBad Homburg, Germany

**Keywords:** Adult stem cells, Cancer, Hepatic stem cells, MicroRNA, Microvesicles, Exosomes

## Abstract

Microvesicles (MVs) play a pivotal role in cell-to-cell communication. Recent studies demonstrated that MVs may transfer genetic information between cells. Here, we show that MVs derived from human adult liver stem cells (HLSC) may reprogram in vitro HepG2 hepatoma and primary hepatocellular carcinoma cells by inhibiting their growth and survival. In vivo intratumor administration of MVs induced regression of ectopic tumors developed in SCID mice. We suggest that the mechanism of action is related to the delivery of microRNAs (miRNAs) from HLSC-derived MVs (MV-HLSC) to tumor cells on the basis of the following evidence: (a) the rapid, CD29-mediated internalization of MV-HLSC in HepG2 and the inhibition of tumor cell growth after MV uptake; (b) the transfer by MV-HLSC of miRNAs with potential antitumor activity that was downregulated in HepG2 cells with respect to normal hepatocytes; (c) the abrogation of the MV-HLSC antitumor effect after MV pretreatment with RNase or generation of MVs depleted of miRNAs; (d) the relevance of selected miRNAs was proven by transfecting HepG2 with miRNA mimics. The antitumor effect of MV-HLSC was also observed in tumors other than liver such as lymphoblastoma and glioblastoma. These results suggest that the delivery of selected miRNAs by MVs derived from stem cells may inhibit tumor growth and stimulate apoptosis. Stem Cells*2012;30:1985–1998*

## INTRODUCTION

Several studies suggest that the embryonic microenvironment may favor reprogramming of tumor cells toward a more benign phenotype [1, 2]. Whether adult stem cells may also have the capability to reprogram the tumor phenotype remains controversial. Bone marrow-derived mesenchymal stem cells (MSCs) were shown under certain circumstances to inhibit tumor growth in a model of Kaposi's sarcoma [3] and of hepatoma [4, 5]. On the other hand, MSC may favor tumor escape by inducing immunosuppression [6, 7].

We recently identified a multipotent human adult liver stem cell (HLSC) population that expresses mesenchymal and embryonic stem cells markers and is partially committed to the hepatic lineage [8, 9]. Microvesicle (MV)-HLSC were found to transfer genetic information to target cells [9, 10]. MVs include a heterogeneous population of vesicles released as exosomes from the endosomal compartment or as shedding vesicles from the cell surface of different cell types [11–13]. It is now recognized that MVs constitute an integral part of the intercellular microenvironment [12, 14, 15]. The broad spectrum of biological activities displayed by MVs candidate them to a pivotal role in cell-to-cell communication. This notion is based on the observation that MVs released from a given cell type may interact through specific receptor–ligand with target cells. After interaction, MVs may directly stimulate the target cells or may transfer various bioactive molecules including mRNAs and microRNAs (miRNAs) from the cell of origin [16–20]. Recently, Balaj et al. [21] found that tumor MVs contain also retrotransposon elements and amplified oncogene sequences. Therefore, MVs may induce epigenetic changes in target cells by delivering specific genetic information. Ratajczak et al. [22] demonstrated that MVs derived from murine embryonic stem cells may reprogram adult hematopoietic stem/progenitor cells. We found that MVs derived from human endothelial progenitors, MSCs, or HLSC can act as a vehicle for exchange of functional mRNAs among cells leading to activation of regenerative programs in differentiated cells both in vitro and in vivo [23, 24, 9]. Valadi et al. [25] showed that exosomes released from mast cells contained specific subset of miRNAs. Yuan et al. [26] demonstrated that miRNAs may be transferred from embryonic stem cells to mouse embryonic fibroblasts via MVs. We recently characterized miRNAs shuttled by HLSC-derived MVs (MV-HLSC) showing that they may potentially affect development, cell fate, differentiation, catalytic activities, and metabolic processes [10].

The aim of this study was to investigate whether MV-HLSC may influence the growth of a hepatoma both in vitro and in vivo. We evaluated in vitro the effect of MVs on cell proliferation and apoptosis resistance of HepG2 hepatoma cell line and of four hepatocellular carcinoma cell (HCC) lines. In vivo, we evaluated whether MV-HLSC induced the tumor regression in an ectopic model of transplantation in SCID mice. In addition, by silencing Dicer in HLSC or using selected miRNA inhibitors, we evaluated the role of miRNAs, shuttled by MV-HLSC, in the suppression of tumor growth.

## MATERIALS AND METHODS

### Cells

HLSC were isolated from human cryopreserved normal hepatocytes obtained from Lonza (Basel, Switzerland, http://www.lonza.com), characterized and cultured as previously described [8]. Normal human hepatocytes and human fibroblasts (Lonza) were cultured as described [9, 24]. Hepatoma cell line HepG2 (American Type Culture Collection, Rockville, MD, http://www.atcc.org) were cultured in Dulbecco's modified Eagle's medium (DMEM) containing 10% fetal bovine serum (FBS). Primary hepatoma cell lines were generated in our laboratory from tumor specimens of four patients with histopathological diagnosis of HCC undergoing surgical resection with informed consent at the Liver Transplantation Center of Molinette Hospital, Turin. HCC cells were cultured in DMEM, 10% FBS and characterized by immunohistochemistry for the expression of α-fetoprotein, heat shock protein 70, cytokeratin 19, 8, and 18 [27, 28]. Glioblastoma cell line DBTRG (kindly provided by Dr. Oliviero) and T lymphoblastic tumor cell line SupT1 (kindly provided by Dr. Tarella) were cultured in Roswell Park Memorial Institute (RPMI)-1640, 10% FBS, and GlutaMAX (Life Technologies, New York, NY, http://www.lifetech.com).

### Generation of Dicer1 Knockdown HLSC

The knockdown of Dicer was obtained by transfecting HLSC with a Dicer1 shRNA plasmid (DCR-kd HLSC) (sc-40489-SH, Santa Cruz Biotechnology, Santa Cruz, CA, http://www.scbt.com) using lipofectamine (Life Technologies). HLSC transfection with a plasmid coding for a scramble short hairpin RNA (shRNA) sequence was used as control (CTR-A HLSC). Cells were grown in the presence of 1 μg/ml puromycin to select stably transfected HLSC that were tested for Dicer expression. Transfected HLSC were used until sixth passage of culture.

### Cell Transfection

Transfection of miScript miRNA Inhibitors and Mimics to HepG2 was performed using the HiPerFect Transfection method (Qiagen, Valencia, CA, http://www.qiagen.com) according to the manufacturer's protocol.

For HepG2 transfection (8 × 10^3^ cells), 10 nM mimics or 100 nM miScript miRNA inhibitors along with 1 μl HiPerFect Transfection Reagent (all from Qiagen) were used. The day after transfection, HepG2 were cultured in their complete medium (for mimics) or in the presence of 30 μg/ml of MV-HLSC (for miRNA inhibitors) for 72 hours. AllStars negative control siRNA (SCR) or miScript Inhibitor negative control (anti-CTR) were used as controls.

For fibroblast transfection, pCMV-Sport six plasmid carrying the human integrin, β1 (CD29) full-length cDNA sequence was purchased from Open Biosystems (Lafayette, CO, http://www.openbiosystems.com). Subconfluent fibroblasts (6 × 10^5^ cells per 100 microliters) were harvested and resuspended in Nucleofector Solution (Lonza). Nucleofection was performed by mixing the cell suspension (100 μl) with the plasmid (6 μg) using the Nucleofector device (Amaxa) and the preoptimized program (U-23). Immediately following nucleofection, the cells were plated in their complete cultured medium. Nucleofection of fibroblasts in the absence of DNA was used as negative control (fibr-CTR). Control pmaxGFP vector (Amaxa) was used as transfection efficiency control. The day after transfection, fibroblasts were cultured overnight in serum-free medium to collect MVs. Cells and MVs were then used for RNA and protein isolation and for the in vitro assays.

### Isolation of MVs

MVs were obtained from supernatants of HLSC cultured overnight in α-Minimum Essential Medium (MEM) with 2% of MV-depleted FBS by differential centrifugation and ultracentrifugation at 100,000*g* as previously described [23]. MVs were also isolated from fibroblasts as previously described [24]. To trace MVs, HLSC were labeled with SYTO RNASelect green fluorescent cell stain (Life technologies), MVs were then collected and labeled with PKH26 dye (Sigma, St. Louis, MO, http://www.sigmaaldrich.com) [23]. Fluorescence-activated cell sorting (FACS) analysis on MVs was performed as previously described [9].

In selected experiments, MV-HLSC were treated with 5 U/ml RNase (Life technologies) for 3 hours at 37°C; the reaction was stopped by addition of RNase inhibitor (Life Technologies) and MVs were washed by ultracentrifugation. Size and distribution of MV after RNase treatment were evaluated by nanoparticle tracking analysis (NTA) using NanoSight LM10 instrument (NanoSight Ltd., Amesbuty, U.K., http://www.nanosight.com) equipped with the NTA 2.0 analytic software [29]. RNase did not affect surface and protein expression of MV as previously reported [24].

### RNA Extraction and Quantitative Real Time Reverse Transcriptase Polymerase Chain Reaction

Total RNA was isolated using the mirVana RNA isolation kit (Life Technologies), quantified spectrophotometrically (Nanodrop ND-1000, Wilmington, DE, http://www.nanodrop.com) and submitted to capillary electrophoresis on Agilent 2100 bioanalyzer (Agilent Tech. Inc., Santa Clara, CA, http://www.agilent.com). Quantitative reverse transcriptase polymerase chain reaction (qRT-PCR) for gene expression analysis was performed as previously described [30]. Sequence-specific oligonucleotide primers used (MWG-Biotech AG, Ebersberg, Germany, http://www.mwg-biotech.com) were listened in Supporting Information Table 1.

miRNA expression was analyzed using the miScript Reverse Transcription Kit and miScript SYBR Green PCR Kit (Qiagen) on a 48-well StepOne Real Time System (Life Technologies) as previously described [10]. miRNA-specific primers to hsa-miR-451, -223, -24, -125b, -31, -21, -122, -16-1, and -410 were used in separate reactions. The RNU48 small nucleolar RNA was used as control. miRNA and mRNA comparison between samples was calculated on relative expression data normalized using GAPDH and 18S, as endogenous controls. Fold change expression with respect to controls was calculated for all samples.

For RNase-treated MVs, fold change in miRNA expression was calculated based on cycle threshold (*C*_t_) differences between treated and untreated MVs (2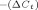) loading the same quantity of RNA during the reverse transcription procedure.

### Incorporation of MVs in Cancer Cells and miRNA Transfer

Incorporation of MVs into tumor cell lines was evaluated by FACS analyses and confocal microscopy after incubation with 50 μg/ml of PKH-26-labeled MVs for 1 hour at 37°C. In selected experiments, MVs were preincubated with blocking antibodies (1 μg/ml) anti-α_4_ integrin, -α_6_ integrin (Biolegend, San Diego, CA, http://www.biolegend.com), anti-CD44 and anti-CD29 (BD Pharmingen, San Jose, CA, http://www.bdbiosciences.com) to inhibit MV incorporation by target cells.

To analyze miRNA transfer from MVs, HepG2 (6 × 10^5^ cells per well) were preplated in a 65 cm^2^ Petri dish and stimulated with 30 μg/ml of MV-HLSC. Cells were coincubated with MVs and a transcription inhibitor, α-amanitin (Sigma, 50 μg/ml) or with α-amanitin alone [31] to inhibit transcriptional activation induced by MVs. miRNA transfer to HepG2 was analyzed by qRT-PCR, at different time points (6, 16, and 24 hours) as previously described [10]. The difference in *C*_t_ values between α-amanitin-treated cells in the absence and in the presence of MVs at each experimental time point was measured; a positive value indicated transfer.

### Proliferation and Apoptosis Assays

DNA synthesis was detected as incorporation of 5-bromo-2-deoxyuridine (BrdU) using an enzyme-linked immunosorbent assay kit (Chemicon, Temecula, CA, http://www.chemicon.com). Apoptosis was evaluated using the terminal dUTP nickend labeling assay (TUNEL, ApopTag, Merck Millipore, Billerica, MA, http://www.millipore.com). Vincristine (10 ng/ml) and doxorubicin (50 ng/ml) were used as positive control of apoptosis. The effect of MV-HLSC on normal human hepatocytes was evaluated by treatment with 5 mM D-Galactosamine (GalN), a hepatocyte-specific apoptotic stimulus [32].

### Western Blot Analysis

After cellular lysis, 100 μg of proteins (Bradford) was subjected to 10% SDS-polyacrylamide gel electrophoresis under reducing conditions, electroblotted onto nitrocellulose membrane filters, and developed with enhanced chemiluminescence (ECL) detection reagents (GE Healthcare Europe GmbH, Freiburg, Germany, http://www.gehealthcare.com) [10]. As primary antibodies, mouse anti-dihydrofolate reductase (DHFR, sc-74593), anti-cyclin D1 (sc-20044), anti-CD29 (sc-71392) and anti-Actin (sc-8432), rabbit anti-E2 transcription factor-2 (E2F-2, sc-22821), anti-BCL-2 (sc-783), anti-XBP-1 (sc-7160), anti-BCL-XL (sc-634), and goat anti-Dicer (sc-25117) (Santa Cruz Biotechnology) were used. Primary antibodies were detected using appropriate HRP-secondary antibodies (Thermo Scientific, MA, http://www.thermoscientific.com).

### Immunofluorescence

Indirect immunofluorescence of HepG2 cell line or on 5-μm paraffin sections of HepG2 tumors was performed by staining with the following antibodies: mouse anti-multidrug resistance protein 1 (MDR1, MAB4334, Chemicon), rabbit anti-ras-related protein 14 (RAB14, ab28639, Abcam, Cambridge, U.K., http://www.abcam. com) and anti-E2F-2 (Santa Cruz) and goat anti-macrophage migration inhibitory factor (MIF, AF-289-PB, R&D Systems, Minneapolis, MN, http://www.rndsystems.com). Paraffin sections were subject to antigen retrieval, washed and labeled with primary antibodies. Primary antibodies were then detected using appropriate secondary antibodies, Alexa Fluor 488 or Texas Red IgG (Molecular Probes, Leiden, The Netherlands, http://probes.invitrogen.com). Omission of the primary antibodies or substitution with nonimmune rabbit IgG was used as controls. Confocal microscopy was performed using a Zeiss LSM5 Pascal confocal microscope (Carl Zeiss Int., Oberkochen, Germany, http://www.zeiss.com). Hoechst 33258 dye (Sigma) was added for nuclear staining.

### In Vivo Tumor Model

Experiments were performed in accordance with the national guidelines and regulation and were approved by the Ethic Committing of the University of Turin. Male SCID mice (4–5 weeks old, Charles River Laboratories, Wilmington, MA, http://www.criver. com) received on day 0, an injection (3 × 10^6^) of tumor cells in serum-free DMEM with Matrigel at a 1:1 ratio. Cell suspension was injected in a total volume of 0.2 ml into the left inguinal area of the SCID mice. Treatments started when tumors reached the volume of approximately 15 mm^3^. Intratumor injection of 100 μg of MVs in a volume of 20 μl or the same volume of vehicle alone was performed weekly for 3 weeks. In select experiments, the day before MV injection, an intratumor injection of select miRNA inhibitors (1.5 nmol/20 μl) was administrated. Tumor mass was determined every 3 days by caliper, measurement in two perpendicular diameters and calculated using the formula 1/2*a* × *b*^2^, where *a* stands for the long diameter and *b* is the short diameter. Mice were sacrificed and tumors were collected for further analysis.

### Morphological Studies

Tumors were frozen in Optimum Cutting Temperature (OCT) or fixed in 10% buffered neutral formalin. Specimens were routinely processed, embedded in paraffin, sectioned at 5 μm, and stained with hematoxylin and eosin (H&E) for microscopic examination. Immunohistochemistry for detection of proliferation and apoptosis was performed using the anti-PCNA monoclonal antibody (Santa Cruz) or TUNEL, respectively, as previously described [9].

### Statistical Analysis

All data of different experiments were expressed as mean ± SD. Statistical analysis was performed by ANOVA with Newmann-Keuls multicomparison or Dunnett's post hoc tests or by Student's *t* test as appropriate. Two-tailed *p* value <.05 was considered statistically significant.

## RESULTS

### MV-HLSC Induced In Vitro Apoptosis and Decreased Proliferation of HepG2 Cells

**Figure 1:**
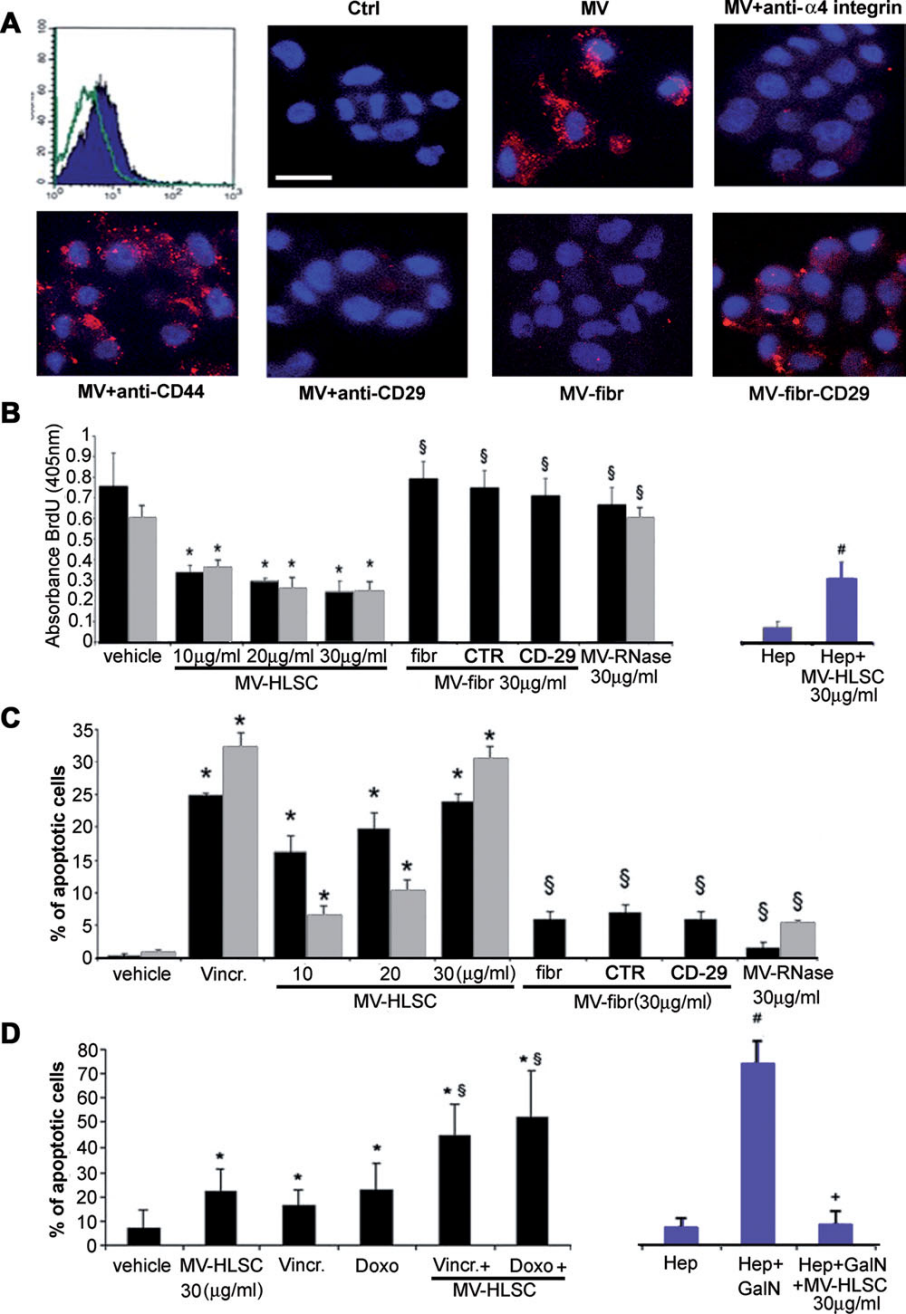
Incorporation of MV-HLSC in hepatoma cells inhibits proliferation and induces apoptosis. (**A**): PKH26-labeled MV-HLSC, or MV-fibr, or MV-fibr-CD29 internalization in HepG2 detected by confocal microscopy in the presence or absence of blocking antibodies against α4 integrin, CD29, and CD44 and by fluorescence-activated cell sorting (FACS) analyses (filled curve: MV-HLSC internalization by HepG2). Kolmogrov-Smirnov statistical analyses of MVs versus Ctrl: *p* < .001. Three experiments were performed with similar results. Scale bar = 10 μm. (**B**): Analysis of HepG2 (black columns) and hepatocellular carcinoma cell (HCC [gray columns]) proliferation by BrdU internalization after 48 hours in the presence of vehicle alone (vehicle) or of different doses of MV-HLSC, MV-fibr, or MV-fibr-CTR, or MV-fibr-CD29, or MV-RNase. Proliferation was evaluated on normal human Hep (blue columns) in the presence of vehicle alone or of MV-HLSC. (**C**): Analysis of HepG2 (black columns) and HCC (gray columns) apoptosis by terminal dUTP nickend labeling assay (TUNEL) assay. Cells were incubated with vehicle, vincristine, or different doses of MV-HLSC, or MV-fibr, or MV-fibr-CTR, or MV-fibr-CD29, or MV-RNase. Results in (B) and (C) are expressed as mean ± SD of six different experiments. (B and C): *, *p* < .05 versus vehicle; ^§^, *p* < .05 versus MV-HLSC treatments. (**D**): Apoptosis of HepG2 coincubated with MV-HLSC and vincristine or Doxo was evaluated. To analyze the antiapoptotic effect of MV-HLSC on normal human Hep (blue columns), apoptosis was induced by 5 mM GalN. Results are expressed as mean ± SD of six different experiments. *, *p* < .05 versus vehicle; ^§^, *p* < .05 versus MV-HLSC, vincristine or Doxo alone; #, *p* < .05 versus Hep; +, *p* < .05 versus Hep+GalN. Abbreviations: BrdU, 5-bromo-2-deoxyuridine; Doxo, doxorubicin; GalN, D-galactosamine; Hep, hepatocytes; HLSC, human adult liver stem cell; MV, microvesicles; MV-HLSC, HSLC-derived MV; MV-fibr, MVs derived from fibroblasts; MV-fibr-CD29, MVs derived from transfected fibroblasts overexpressing the CD29; MV-RNase, RNase-inactivated MVs.

We previously demonstrated that MV-HLSC expressed several adhesion molecules also present on HLSC plasma membrane such as α4 integrin, CD29, and CD44 [9]. To investigate the role of adhesion molecules expressed on MV surface in the incorporation into target cells, MV-HLSC were preincubated with blocking antibodies (1 μg/ml) against α4 integrin, α6 integrin, CD29, or CD44 before the incubation with the cells. MV-HLSC labeled with PKH26 dye were incorporated by HepG2 after 1 hour of incubation at 37°C as shown by confocal microscopy and FACS analysis (Fig. 1A). Pretreatment with anti-α4 integrin and anti-CD29 blocking antibodies inhibited MV-HLSC incorporation in HepG2. Blockade of CD44 did not inhibit the incorporation of MV-HLSC in HepG2 (Fig. 1A). The anti-α6 integrin blocking antibody, used as negative control, was unable to prevent MV-HLSC internalization in HepG2 since MV-HLSC do not express this integrin [9].

MV-HLSC inhibited proliferation (Fig. 1B) and induced apoptosis in HepG2 and HCC cell lines in a dose-dependent manner (Fig. 1C). On the contrary, in normal human hepatocytes that internalize MV-HLSC, the latter slightly stimulated hepatocyte proliferation (Fig. 1B) and were protective in D-galactosamine (GalN)-induced apoptosis (Fig. 1D). The apoptotic effect induced by MV-HLSC on HepG2 was comparable to that of vincristine and doxorubicin used as positive controls (Fig. 1D). When MV-HLSC were coincubated with these chemotherapeutic agents, a significant enhancement of apoptosis was observed (Fig. 1D). The effect of MV-HLSC was specific since the MVs derived from fibroblasts (MV-fibr) were ineffective (Fig. 1B, 1C). MV-fibr lack CD29 [24] and were only slightly internalized by HepG2 (Fig. 1A). The absence of CD29 in MVs from fibroblasts was the main cause of reduced internalization. In fact MVs derived from transfected fibroblasts overexpressing the CD29 (MV-fibr-CD29; Supporting Information Fig. 1A) were internalized by HepG2 in a manner comparable to that of MV-HLSC (Fig. 1A). However, the enhanced internalization of MV-fibr-CD29 did not affect HepG2 cell proliferation and apoptosis (Fig. 1B, 1C).

When MV-HLSC were treated with high, unphysiological concentration of RNase, that was previously shown to degrade the MV-shuttle RNA [23] without affecting their structure (Supporting Information Fig. 1B), the effect of MV-HLSC on proliferation and apoptosis of HepG2 and HCC cell lines was significantly reduced (Fig. 1B, 1C). These results suggest that the biological effects of MV-HLSC on HepG2 were mediated by the transfer of RNAs.

### MV-HLSC Reduced In Vivo Growth of HepG2 Tumors in SCID Mice

**Figure 2:**
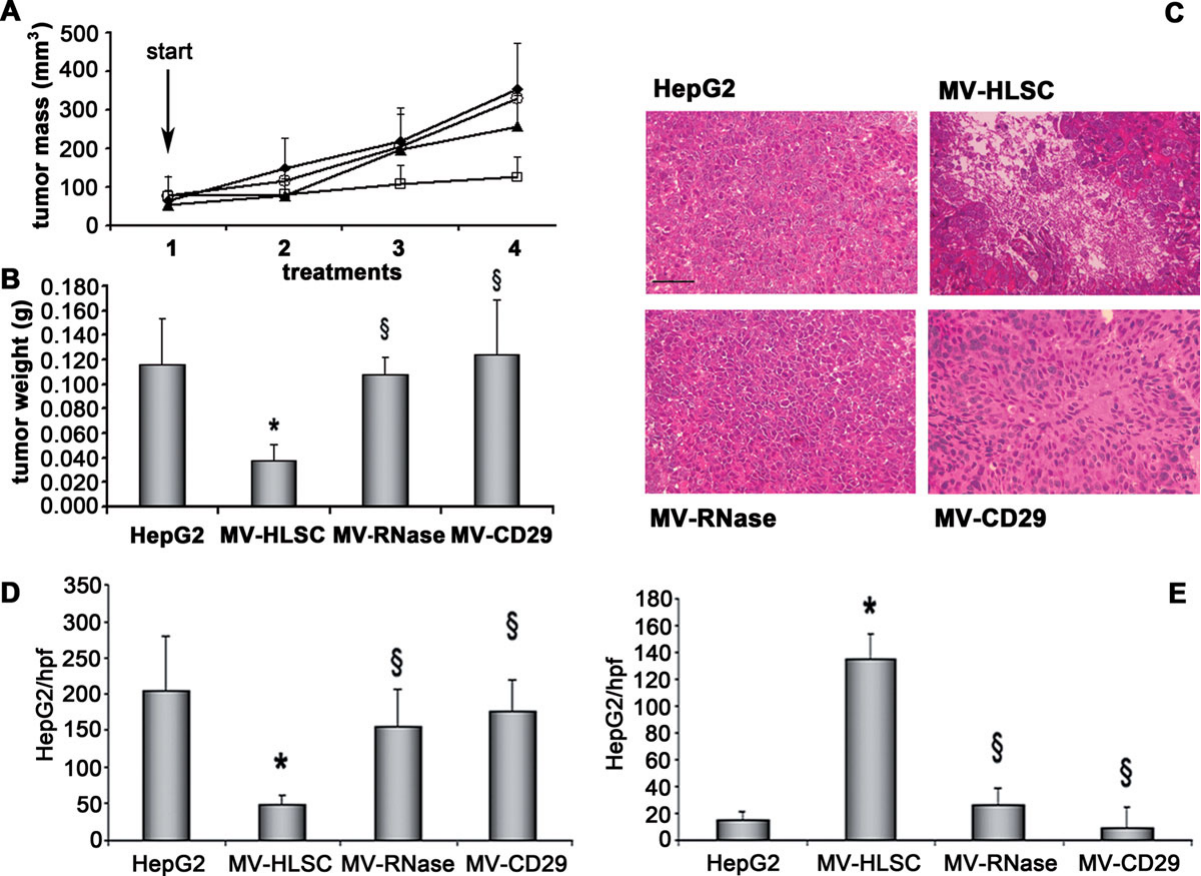
In vivo antitumor effect of MV-HLSC. (**A**): Tumor growth in SCID mice injected subcutaneously with 3 × 10^6^ HepG2 and after 1 week treated weekly with vehicle (♦, HepG2) or 100 μg of MV-HLSC (□), with MV-RNase (▴) or with CD29 blocking antibody-treated MV-HLSC (○, MV-CD29). (**B**): Tumor weights after excision from severe combined immunodeficiency (SCID) mice at sacrifice. Results are expressed as mean ± SD of 12 mice for experimental group. *, *p* < .05 versus HepG2; ^§^, *p* < .05 versus MV-HLSC. (**C**): Representative micrographs of H&E staining of tumor sections from SCID mice treated with vehicle alone (HepG2) or with MV-HLSC, MV-RNase, and MV-CD29. Large areas of necrosis were visible in tumors treated with MV-HLSC. Scale bar = 50 μm. (**D**): Quantification of Proliferating Cell Nuclear Antigen (PCNA)-positive cells/hpf was calculated. (**E**): Quantification of apoptotic cells/hpf was calculated. Data are expressed as mean ± SD of 12 mice for experimental group. *, *p* < .05 versus HepG2; ^§^, *p* < .05 versus MV-HLSC. Abbreviations: HLSC, human adult liver stem cells; hpf, high power field; MV, microvesicles; MV-HSLC, HSLC-derived MV; MV-RNase, RNase-inactivated MVs; MV-CD29, MVs pretreated with anti-CD29 blocking antibody.

When injected subcutaneously in SCID mice, HepG2 formed tumors that became palpable after 1 week. Tumor growth was monitored for 4 weeks. Treatment with HLSC-derived MVs was started after 1 week by direct injection within the tumor of 100 μg of MVs in a volume of 20 μl weekly for 3 weeks. As shown in Figure 2 A and 2B, the increase in size and weight of HepG2 tumors was significantly reduced in mice injected with MV-HLSC with respect to controls injected with vehicle alone (HepG2) or to mice injected with RNase-inactivated MVs (MV-RNase) (Fig. 2A–2C). To evaluate the role of CD29 in MV internalization by HepG2, in selected experiments, we pretreated MVs with anti-human CD29 blocking antibody (MV-CD29). The CD29 blockade abrogated the in vivo effects mediated by MVs (Fig. 2A–2C). The proliferation of HepG2 within the tumor mass detected by PCNA, was significantly reduced in MV-HLSC treated tumors with respect to control mice injected with vehicle alone, with MV-RNase or with MV-CD29 (Fig. 2D; Supporting Information Fig. 2A). Apoptosis detected by TUNEL was significantly enhanced in mice treated with MV-HLSC with respect to controls injected with vehicle alone or to mice injected with MV-RNase or with MV-CD29 (Fig. 2E; Supporting Information Fig. 2B).

### Role of MV-Shuttled miRNAs in the Antitumor Activity of MV-HLSC

**Figure 3:**
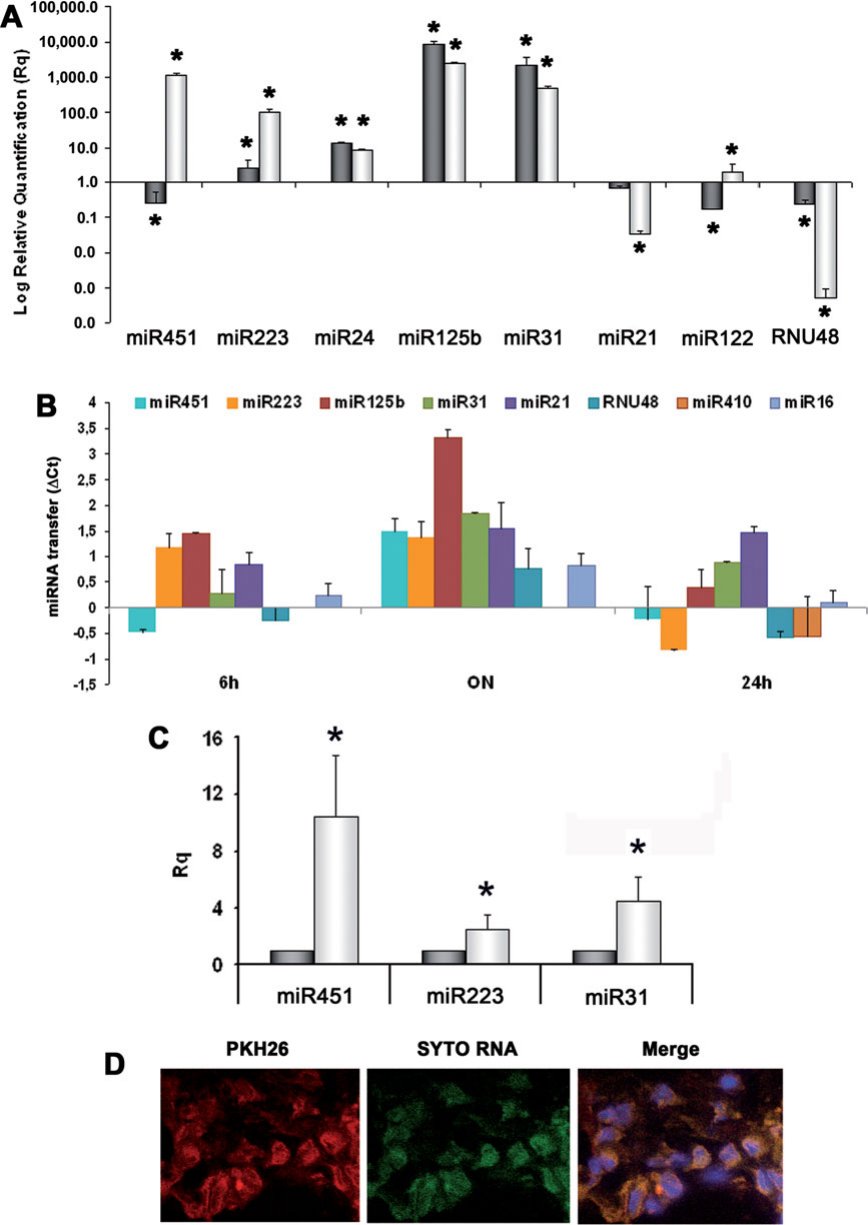
MV-mediated transfer of miRNAs with potential antitumor activity to HepG2. (**A**): The expression of miR451, miR223, miR24, miR125b, miR31, miR21, miR122, and RNU48 was evaluated by quantitative reverse transcriptase polymerase chain reaction (qRT-PCR) in HLSC (gray bars) and MV-HLSC (white bars) with respect to HepG2. Data were expressed as log of Rq, normalized to GAPDH mRNA and to one for HepG2. Some of these miRNAs were significantly enriched in MVs with respect to their relative human adult liver stem cell (HLSC) and were less expressed or not detectable in HepG2. Data are the mean ± SD of four experiments. *, *p* < .05 versus HepG2. (**B**): HepG2 were incubated with HLSC-derived MV (MV-HLSC) 6 hours, overnight (ON, 16 hours), and 24 hours (h), and the transfer of selected miRNAs was evaluated by qRT-PCR. The difference in *C*_t_ values between HepG2 treated with vehicle or stimulated with MV-HLSC was shown for each miRNA. The RNU48 and the miR410, that was not present in MVs, were used as controls. Data are the mean ± SD of four experiments. (**C**): Relative expression of miR451, miR223, and miR31 in MV-HLSC (white bar) with respect to MVs derived from fibroblasts (MV-fibr [gray bar]) evaluated by qRT-PCR. Data were normalized to GAPDH mRNA, to one for MV-fibr and expressed as mean ± SD. Three experiments were conducted with similar results. *, *p* < .05 versus MV-fibr. (**D**): Representative micrographs showing the in vivo internalization in tumors of MV-HLSC labeled with PKH26 and SYTO RNA that stain MV membrane and RNA content, respectively. Three experiments were performed with similar results. Abbreviations: miRNA, micro RNA; Rq, relative quantification.

We recently demonstrated the presence of enriched miRNAs in MVs released by HLSC [10]. Among miRNAs present in MV-HLSC, we detected several miRNAs with potential antitumor activity including miR451, miR223, miR24, miR125b miR31, and miR122 (Fig. 3A). Some of these miRNAs were significantly enriched in MV-HLSC with respect to their relative HLSC suggesting a specific compartmentalization within MVs. Moreover, these miRNAs were not detectable or were expressed at low levels in HepG2 (Fig. 3A). On the contrary, miR21 was not enriched in MV-HLSC but downregulated with respect to HepG2. The levels of different miRNAs relative to each other in MV-HLSC are shown in Supporting Information Figure 1D.

To investigate whether MVs may transfer miRNAs to target cells, HepG2 were incubated with MV-HLSC in the presence of α-amanitin to inhibit transcriptional activation. The transfer of selected miRNAs was evaluated by qRT-PCR. The variation in cycle threshold (*C*_t_) values in HepG2 stimulated with MV-HLSC in the presence of α-amanitin was evaluated with respect to HepG2 treated with α-amanitin alone. The accumulation of miRNAs into HepG2 treated with MVs peaked after overnight stimulation (16 hours) to decrease at 24 hours (Fig. 3B). The miR-410, not present in MV-HLSC and used as a negative control, was not detected in HepG2 after MV incorporation. This suggested that the increase in specific miRNA content was due to their transfer from MVs to the HepG2. MVs from fibroblasts, used as control, contained significant less amount of tumor suppressive miR451, 223, and 31, than MV-HLSC (Fig. 3C).

The RNA shuttled by MVs into HepG2 was also evaluated in vivo. MVs isolated from HLSC labeled with SYTO RNA dye that tagged cell RNA were then stained with PKH26 and injected intratumor. The analysis of excised tumors showed the colocalization of both dyes, suggesting the incorporation of RNA-containing MV into HepG2 tumors (Fig. 3D).

**Figure 4:**
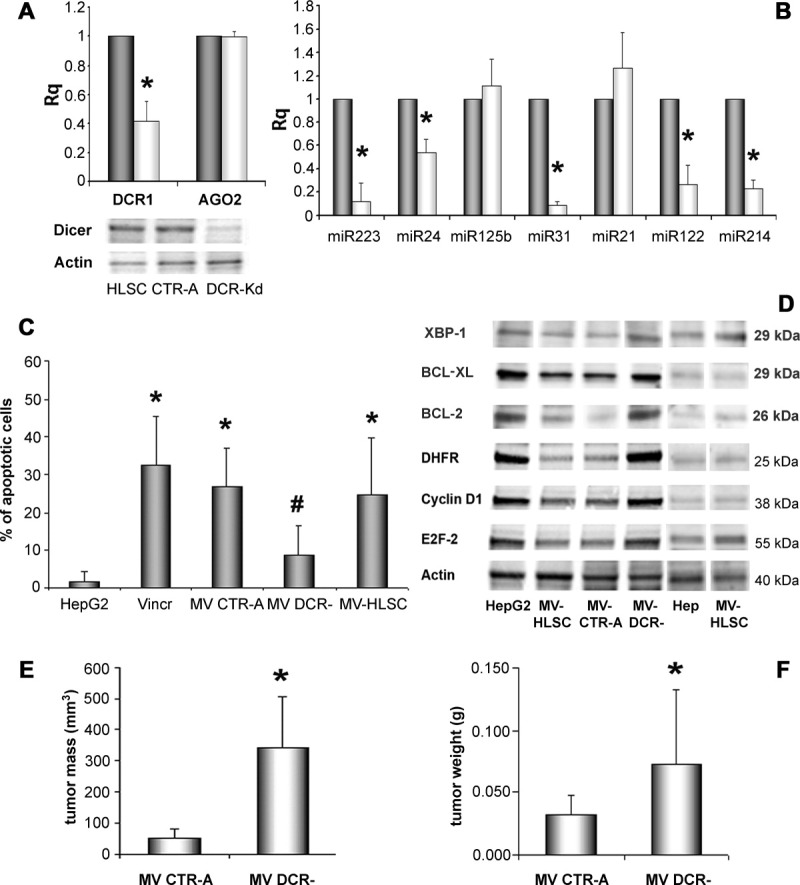
Effect of MVs derived from DCR-kd HLSC on in vitro HepG2 apoptosis and in vivo tumor growth. (**A**): Quantitative reverse transcriptase polymerase chain reaction (qRT-PCR) analysis of DCR1 and AGO2 transcripts in DCR-Kd (white bar) and CTR-A (gray bar). Western blot of Dicer protein expression (lower panel). Actin was used as endogenous control. *, *p* < .05 versus CTR-A. (**B**): Analysis of miRNAs in MV DCR− (white bar) or in MV CTR-A (gray bar) evaluated by qRT-PCR. *, *p* < .05 versus MV CTR-A. Data in (A) and (B) are representative of three different experiments. Data were normalized to GAPDH mRNA and to one for CTR-A and MV CTR-A. (**C**): Analysis of HepG2 apoptosis by terminal dUTP nickend labeling assay (TUNEL) assay. Cells were incubated with vehicle alone (HepG2), with 10 ng/ml vincristine, or with 30 μg/ml of MV CTR-A, MV DCR−, or control MV-HLSC. Results are expressed as mean ± SD of five different experiments. *, *p* < .05 versus HepG2; #, *p* < .05 versus MV CTR-A. (**D**) Expression of XBP-1, BCL-XL, BCL-2, DHFR, cyclin D1, E2F-2, and Actin was evaluated by Western blot in HepG2 treated with vehicle alone (HepG2) or with 30 μg/ml of MV-HLSC, MV CTR-A, or MV DCR−, and in human hepatocytes treated with vehicle alone (Hep) or with 30 μg/ml of MV-HLSC. Three experiments were conducted with similar results. (**E, F**): HepG2 were injected subcutaneously in severe combined immunodeficiency (SCID) mice and treated weekly with 100 μg of MV CTR-A or of MV DCR−. Measure of tumor mass (E) and weight (F) was evaluated at sacrifice. Results are expressed as mean ± SD of five mice for experimental group. *, *p* < .05 versus MV CTR-A. Abbreviations: AGO2, Argonaute 2 transcript; CTR-A, scramble transfected control HLSC; DCR1, Dicer 1; DCR-kd, Dicer knockdown; Hep, hepatocytes; HLSC, human adult liver stem cell; MV-HLSC, HSLC-derived MV; MV CTR-A, MVs from CTR-A transfected HLSC; MV DCR−, MVs from DCR-kd HLSC; Rq, relative quantification.

To evaluate whether miRNAs shuttled by MV-HLSC were relevant for the tumor suppression, we generated MVs from Dicer knockdown HLSC (DCR-Kd HLSC). As shown in Figure 4A, Dicer was selectively downregulated at mRNA and protein levels in DCR-Kd HLSC but not in scramble transfected control HLSC (CTR-A HLSC). The specificity of downregulation of Dicer was indicated by the unaltered expression of the Argonaute 2 transcript. MVs released from DCR-Kd HLSC (MV DCR−), but not from CTR-A HLSC (MV CTR-A), showed a significant reduction of miR223, miR24, miR31, miR122, and miR214 as detected by qRT-PCR (Fig. 4B). No modulation in miR21 and miR125b levels was observed.

As shown in Figure 4C, MVs derived from DCR-Kd HLSC, but not from CTR-A HLSC, exhibited a significant reduction of the in vitro proapoptotic effect on HepG2. Moreover, the incubation of HepG2 with MVs derived from HLSC resulted in the reduction of proteins known to be targeted by some of the enriched miRNAs found in MV-HLSC (Fig. 4D). In particular, the following target proteins, involved in cell proliferation, were downregulated: DHFR targeted by miR24 [33], Cyclin D1 targeted by miR223 [34], and E2F-2 targeted by miR31 [35]. Moreover, the following target proteins, involved in resistance to apoptosis were downregulated: BCL-XL, targeted by miR122, BCL-2, targeted by miR122 [36], and in less extent XBP-1, targeted by miR214 [37]. No downregulation of proteins involved in cell proliferation and apoptosis resistance was observed on normal human hepatocytes treated with MV-HLSC (Fig. 4D). Actin was used as control. MV DCR−, but not MV CTR-A, did not induce downregulation of these target proteins. In addition, MV DCR− did not significantly inhibit the growth of HepG2 tumors in SCID mice (Fig. 4E, 4F).

### In Vitro Biological Effect of Specific miRNA Inhibitors

**Figure 5:**
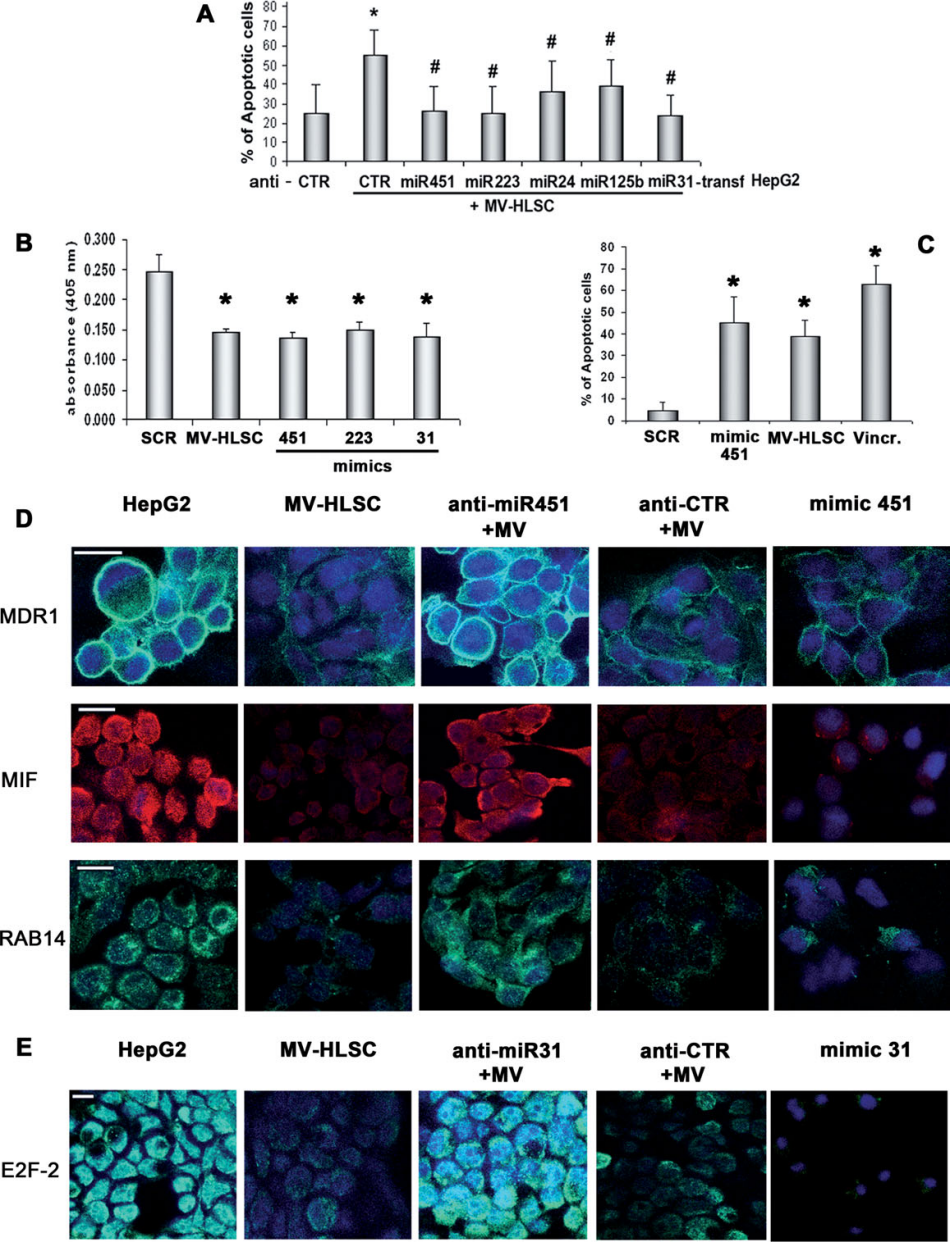
Effect of specific microRNA (miRNA) inhibitors and of miRNA mimics on in vitro apoptosis and on modulation of target proteins. (**A**): terminal dUTP nickend labeling assay (TUNEL) assay of HepG2 transfected with specific miRNA inhibitors and with anti-CTR inhibitor and treated with MV-HLSC. Results are expressed as mean ± SD of three different experiments. *, *p* < .05 versus anti-CTR alone; #, *p* < .05 versus anti-CTR+MV-HLSC. (**B**): The antiproliferative effect of MV-HLSC on HepG2 was compared with the effect of miR451, miR223, and miR31 mimics by 5-bromo-2-deoxyuridine incorporation. (**C**): The proapoptotic effect of miR451 mimic on HepG2 was evaluated by TUNEL assay and compared with that of MV-HLSC and vincristine. Results are expressed as mean ± SD of six different experiments. *, *p* < .05 versus SCR. (**D**): Representative micrographs showing the downregulation of MDR1, MIF, and RAB14 targeted by miR451 in HepG2 incubated with MV-HLSC or transfected with mimic 451. Transfection of HepG2 with anti-miR451 (anti-miR451+MV) but not with anti-CTR abrogated the target downregulation induced by MV-HLSC. (**E**): Representative micrographs showing the downregulation of E2F-2 targeted by miR31 in HepG2 incubated with MV-HLSC or transfected with mimic 31. Transfection of HepG2 with anti-miR31 (anti-miR31+MV) abrogated the E2F-2 downregulation induced by MV-HLSC. Experiments were repeated three times with similar results. Scale bar = 10 μm. Abbreviations: Anti-CTR, negative control miRNA; CTR, control; MV-HLSC, HLSC derived microvesicles; MDR, multidrug resistance protein 1; RAB14, ras-related protein 14; MIF, migration inhibitory factor; SCR, negative control siRNA transfected HepG2.

To evaluate whether single miRNAs with antitumor activity (miR451, miR223, miR24, miR125b, and miR31) were relevant for the proapoptotic effect of MV-HLSC, we transfected HepG2 with selected miRNA inhibitors (Fig. 5A). HepG2 transfected with a negative control miRNA inhibitor (anti-CTR) did not differ from HepG2 wild-type in sensitivity to apoptosis induced by MV-HLSC. In contrast, HepG2 transfected with selected miRNA inhibitors exhibited a significant reduction of sensitivity to apoptosis induced by MVs (Fig. 5A). This was more significant for miR451, miR223, and miR31. The observation that blockade of individual miRNAs completely abrogated, or significantly reduced, the effect of MV-HLSC suggest that each of these miRNA is a critical component of the puzzle that controls cell proliferation and survival. HepG2 transfection with miR451, miR31, and miR223 mimics, which reproduce mature endogenous miRNAs, inhibited proliferation of HepG2 (Fig. 5B). The miR451 mimic mediated also a proapoptotic effect comparable to that of MV-HLSC (Fig. 5C). In particular, we analyzed the involvement in MV-HLSC antitumor activity of miR31 and miR451, previously described as regulators of proliferation and as tumor suppressors in different cancer cells [35, 38, 39]. As shown in Figure 5D, MDR1, targeted by miR451 [40], was strongly expressed on HepG2 cell membrane. After incubation with MV-HLSC, the expression of MDR1 was markedly reduced (Fig. 5D). When HepG2 were transfected with the miRNA inhibitor, anti-miR451, MVs were unable to downregulate the MDR1 expression, suggesting that miR451 transferred by MVs was biologically active. Transfection with the negative control miRNA inhibitor (anti-CTR) was ineffective. Transfection of HepG2 with anti-miR451 without MV treatment, did not affect MDR1 expression (data not shown). We also observed that the HepG2 stimulation with MV-HLSC induced a downregulation of two other important miR451 targets, MIF and RAB14 (Fig. 5D) [40, 41]. Their downregulation, induced by MVs, was abrogated when HepG2 were transfected with anti-miR451, but not with anti-CTR (Fig. 5D). Similar results were observed for E2F-2 which is targeted by miR31 (Fig. 5E). Furthermore, HepG2 transfection with miR451 and miR31 mimics resulted in MDR1, MIF, RAB14, and E2F-2 downregulation similar to that of MV-HLSC (Fig. 5D, 5E).

The potential relevance of miRNA transfer from HLSC to HepG2 was outlined by the observation that miRNAs with antitumor activity are slightly or not detectable in HepG2 whereas they are highly expressed in normal human hepatocytes (Supporting Information Fig. 3A). On the contrary, miR21, known to act as oncomiR in different tumors [41], was more expressed in HepG2 than in normal human hepatocytes. The differences in miRNA expression were paralleled by a difference in the expression of their specific targets. Supporting Information Figure 3B shows that MDR1, MIF, and RAB14, targeted by miR451, and E2F-2, targeted by miR31, were overexpressed at RNA level in HepG2 with respect to hepatocytes. As shown in Figure 3C, the content of tumor-suppressive miRNAs miR451, 223, and 31, in MVs from fibroblast used as control was significantly lower than that of MV-HLSC. Treatment of MV-HLSC with high concentration of RNase (5 U/ml) significantly decreased the levels of different miRNAs shuttled by MVs (Supporting Information Fig. 1C).

### In Vivo Biological Effect of Anti-miR451 or Anti-miR31 Inhibitors

**Figure 6:**
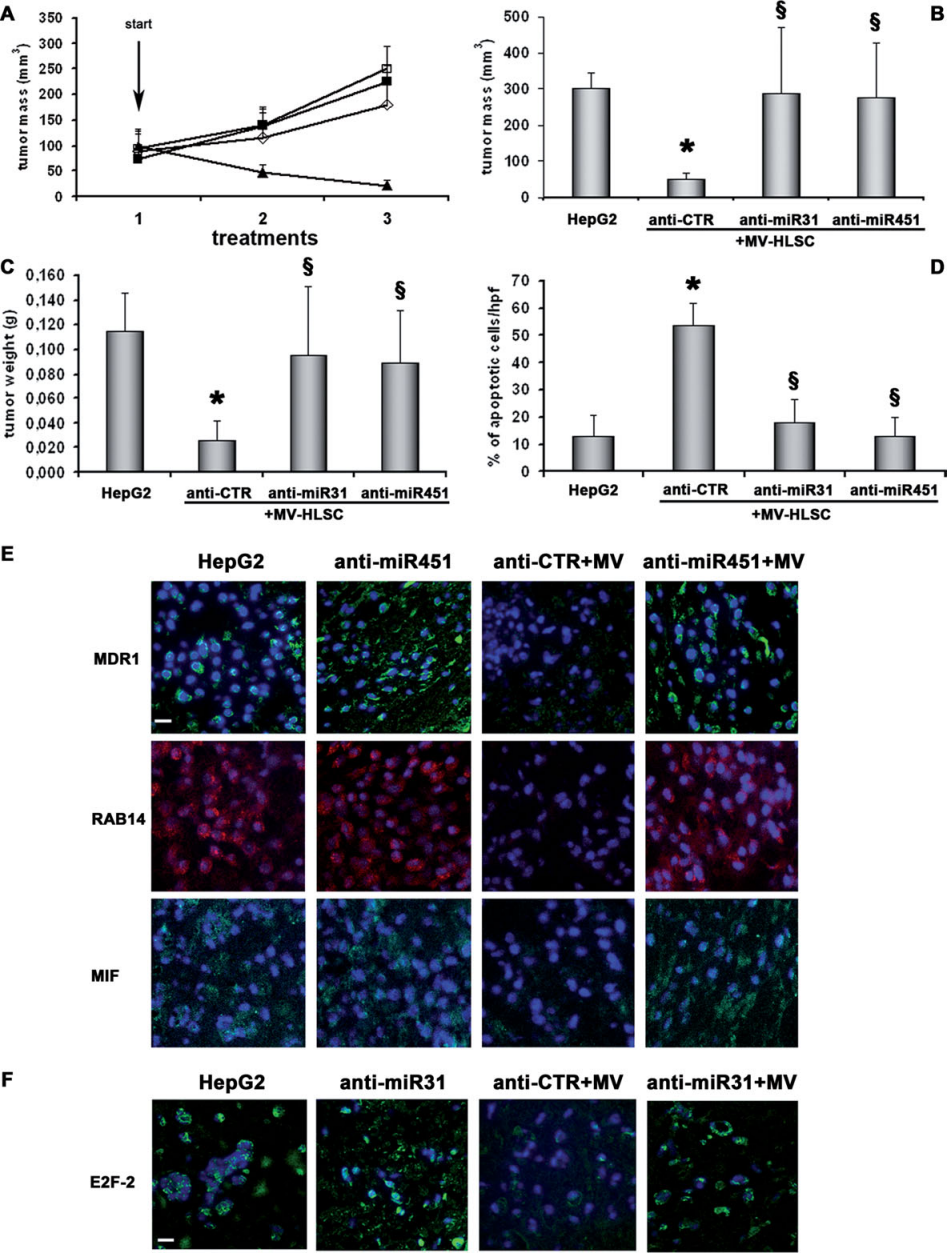
Effect of specific microRNA inhibitors on MV-induced inhibition of tumor growth and on modulation of target proteins. (**A–D**): Tumor growth in severe combined immunodeficiency (SCID) mice injected subcutaneously with 3 × 10^6^ HepG2 and treated weekly with vehicle (▪, HepG2) or with 100 μg of MV-HLSC in the presence of anti-miR31 (⋄) or anti-miR451 (□) or anti-CTR (▴). Tumor mass was determined weekly by caliper (**A**) and by measure of tumor size (**B**) and weight (**C**) at sacrifice. (**D**): Apoptotic cells within the tumors were evaluated by terminal dUTP nickend labeling assay (TUNEL) assay. Results in (B–D) are expressed as mean ± SD of six mice for experimental group. *, *p* < .05 versus HepG2; ^§^, *p* < .05 versus anti-CTR+MV-HLSC. (**E**): Representative micrographs showing the in vivo expression of MDR1, MIF, and RAB14 in HepG2, the downregulation of targets by MV-HLSC (anti-CTR+MV), and the abrogation of downregulation by anti-miR451 treatment (anti-miR451+MV). Treatment with anti-miR451 without MV administration (anti-miR451) did not interfere with protein expression by HepG2. (**F**): Representative micrographs showing the in vivo expression of E2F-2 in HepG2, its downregulation by MV-HLSC treatment in the presence of anti-CTR inhibitor (anti-CTR+MV), and the abrogation of downregulation by anti-miR31 (anti-miR31+MV). Treatment with anti-miR31 without MV administration (anti-miR31) did not interfere with E2F-2 expression by HepG2. Micrographs are representative of six different experiments. Scale bar = 10 μm. Abbreviations: anti-CTR, negative control miRNA; HLSC, human adult liver stem cell; MV-HLSC, HLSC derived MV; MDR, multidrug resistance protein 1; RAB14, ras-related protein 14; MIF, migration inhibitory factor.

The in vivo potential involvement of miR451 and miR31 was evaluated by intratumor administration of anti-miR451 or anti-miR31 or of negative control anti-CTR 24-hour before the administration of MV-HLSC. Experiments performed with a ter 6-carboxyfluorescein (FAM) labeled negative control miRNA inhibitor showed that the intratumor injection is followed by its internalization within the cells (not shown). In mice treated with anti-miR451 and anti-miR31, the inhibition of tumor growth induced by MV-HLSC was significantly less effective than in animals treated with anti-CTR (Fig. 6, A–6C). We observed a reduced apoptosis in tumors treated with MV-HLSC in the presence of the selected anti-miRNAs (anti-miRs) with respect to tumors treated with MVs in the presence of anti-CTR (Fig. 6D). MDR1, MIF, RAB14, and E2F-2 were highly expressed by HepG2 tumors in vivo as detected by immunofluorescence (Fig. 6E, 6F). MV treatment downregulated the in vivo expression of these target proteins. The inhibitory effect of MV-HLSC was abrogated by anti-miR451 and anti-miR31 administration (Fig. 6E, 6F). Treatment with anti-miR451 and anti-miR31 in the absence of MVs did not interfere with target expression by hepatoma tumors.

**Figure 7:**
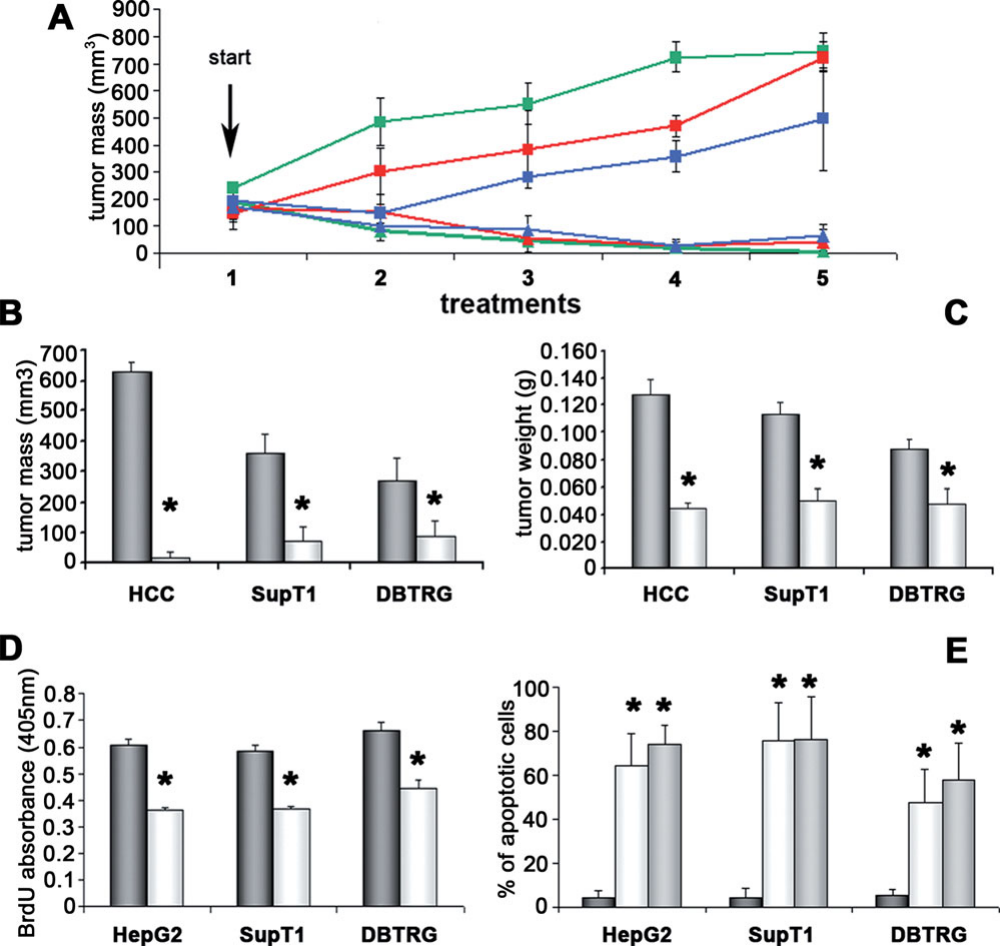
In vitro and in vivo effect of human adult liver stem cell-derived microvesicle (MV-HLSC) on HCC hepatocarcinoma, SupT1 lymphoblastoma, and DBTRG glioblastoma cells. (**A**): Tumor growth in severe combined immunodeficiency (SCID) mice injected subcutaneously with 3 × 10^6^ HCC (▪ green), SupT1 (▪ red), or DBTRG (▪ blue) and treated weekly with vehicle or 100 μg of MV-HLSC (HCC = ▴ green, SupT1 = ▴ red, and DBTRG = ▴ blue). Measure of tumor mass (**B**) and tumor weight (**C**) excised from SCID mice treated with vehicle alone (black bars) or with MV-HLSC (white bars) at sacrifice. Results are expressed as mean ± SD of four mice for experimental group. *, *p* < .05 versus vehicle alone. Effect of MV-HLSC (30 μg/ml) on in vitro proliferation evaluated by BrdU incorporation (**D**) and on apoptosis evaluated by TUNEL (**E**) of HepG2, SupT1, and DBTRG. Vincristine was used as positive control of apoptosis (gray bar). Results in (**D**) and (**E**) are expressed as mean ± SD of six individual experiments. *, *p* < .05 versus vehicle alone. Abbreviations: BrdU, 5-bromo-2-deoxyuridine; HCC, hepatocellular carcinoma cell.

MV-HLSC significantly inhibited also the growth of tumors induced in SCID mice by primary HCCs (Fig. 7A). To evaluate whether the biological effect of MV-HLSC was specific to hepatic tumors, we investigated also SupT1 lymphoblastoma and DBTRG-05MG glioblastoma cells (DBTRG) injected subcutaneously in SCID mice. As shown in Figure 7, MV-HLSC significantly inhibited SupT1 and DBTRG tumor growth (Fig. 7A–7C). This was confirmed in vitro where MVs inhibited proliferation and induced apoptosis of tumor cells (Fig. 7D, 7E).

## DISCUSSION

The results of this study demonstrated that MVs released from HLSC inhibited the growth of hepatoma cells both in vitro and in vivo. We suggest that the mechanism is related to the delivery of miRNAs from MV-HLSC to tumor cells on the basis of the following evidence: (a) the rapid, CD29-mediated internalization of MVs in tumor cells and the inhibition of their growth after MV uptake; (b) the transfer of antitumor MV-HLSC miRNAs that were downregulated in HepG2 cells with respect to normal hepatocytes; (c) the abrogation of the MV-HLSC antitumor effect after the MV pretreatment with RNase, Dicer silencing in HLSC, and the transfection of HepG2 with specific miRNA inhibitors; (d) finally, the relevance of selected miRNAs as proven by transfecting HepG2 with miRNA mimics.

MVs/exosomes have been recently described as new mediators of cell-to-cell communication that may reprogram the target cells through the active transfer of proteins, functional mRNAs, and miRNAs [13]. In particular, the finding that MVs may carry selected patterns of mRNAs and miRNAs have suggested their involvement in genetic exchange among cells [22, 23, 25, 26]. As stem/progenitor cells are an abundant source of MVs, it is conceivable that they play a role in stem cell biology [42, 43]. Stem cell-derived MVs are enriched in miRNAs that can be transferred to other cells. The biological effects of such miRNAs may be different depending on the genetic state of the recipient cells. miRNA expression is known to be deregulated in tumors [44]. In particular, it has been shown a downregulation of miRNAs with tumor suppressor activity and an upregulation of miRNAs that may act as oncogenes [45–47].

In this study, we evaluated whether MV-HLSC may influence the in vitro behavior of HepG2 cells and of primary HCC cells. It is now recognized that upon release MVs may be internalized in target cells through specific receptor–ligand interactions allowing the transfer of specific signals [9]. When incubated with HepG2 or HCC cells, labeled MVs were rapidly internalized. In particular, we demonstrated that the adhesion molecule mainly involved in MV uptake by hepatoma cells was the CD29. Indeed, the CD29 blockade inhibited the MV internalization by tumor cells. The uptake of MV-HLSC by HepG2 and HCC cells in vitro resulted in a significant inhibition of tumor cell growth and stimulation of apoptosis. In vivo treatment with MV-HLSC significantly reduced the growth of HepG2 tumors formed in SCID mice by enhancing tumor apoptosis and reducing proliferation. When MV-HLSC were pretreated with a CD29 blocking antibody, the antitumor effect was abrogated, suggesting a role of surface molecules in the MV uptake by target cells.

We previously suggested that the biological effects observed on normal cells could be ascribed to the transfer by MVs of selected patterns of mRNAs and miRNAs able to activate regenerative programs [9, 10]. This interpretation is strengthened by several other studies that demonstrated that the genetic information transferred by MVs is the main effector of functional and phenotypical changes occurring in recipient cells [10, 25, 26, 48]. Here, MV-HLSC slightly stimulated proliferation and protected from apoptosis normal human hepatocytes at variance with hepatoma cells. The discrepancy in behavior between normal and tumor cells is possibly due to the differential activation of their intracellular pathways. It is well-established that tumor cells are deficient of several miRNAs relevant in the control of cell growth [44]. This is confirmed in our experimental setting by a qRT-PCR comparative analysis of miRNA expression between HepG2 and normal human hepatocytes.

Here, we observed that the antitumor effect of MV-HLSC was mainly dependent on the transfer of selected miRNAs. Therefore it can be hypothesized that MVs released from HLSC may deliver to HepG2 miRNAs able to reprogram these cells to a more benign phenotype both in vitro and in vivo. Among miRNAs present in MV-HLSC [10], several ones were associated with potential antitumor activity, such as miR451, miR223, miR24, miR125b, miR31, miR214, and miR122. Some of these miRNAs were not or only slightly detectable in HepG2 at variance of normal hepatocytes.

Experiments with RNase treatment indicated that a depletion of miRNAs shuttled by MVs was associated with a reduced biological activity both in vitro and in vivo. The relevance of miRNA transfer to HepG2 via MVs was also derived from experiments with MVs obtained from HLSC knockdown for Dicer1, which is critical for the maturation of miRNAs [49–51]. Previous studies of Dicer depletion in mouse embryonic stem cells demonstrated a role of miRNAs in stem cell differentiation and self-renewal capability [52, 53]. Furthermore, Oskowitz et al. recently showed that the miRNA processing machinery is also involved in the osteogenic and adipogenic differentiation of adult human MSC [54].

Silencing Dicer in HLSC resulted in the modulation of different miRNAs, with a significant reduction of the antitumor miR223, miR24, miR31, and miR122 [55] in MVs. The MV DCR− isolated from DCR-Kd HLSC showed a significant reduction of the in vitro proapoptotic activity and the in vivo antitumor effect.

Specific target proteins involved in cell cycle control, were downregulated in HepG2 treated with MV-HLSC. Interestingly, these proteins were targeted by miRNAs present in MV-HLSC and reduced in MV DCR−. When HepG2 were treated with MV DCR−, the reduction of such targets was absent. These experiments indicate that miRNAs shuttled by MV-HLSC were functionally intact.

Moreover, the use of miRNA inhibitors against miR451, miR223, miR24, miR125b, and miR31 on HepG2 reduced the proapoptotic activity induced by MV-HLSC. The most convincing results were obtained with inhibitors of miR31 and 223 that were downregulated also in MV DCR− as well as with the inhibitor of miR451, that has been previously described as a Dicer independent miRNA [56]. Moreover, the miR451 has been shown to regulate drug resistance to chemotherapeutics mediated by MDR1/P-glycoprotein [41]. MV treatment induced a reduction of MDR1 surface expression in HepG2. This observation may explain our results showing a combinatory effect of MV-HLSC and chemotherapeutic agents in the enhancement of apoptosis. HepG2 stimulation with MV-HLSC induced a downregulation of MIF and RAB14, previously shown to be important miR451 targets [39, 40]. The downregulation of miR451 target proteins, induced by MV-HLSC, was abrogated when HepG2 were transfected with anti-451 miRNA inhibitor. Similar results were observed for E2F-2, which is targeted by miR31 [38], indicating the relevance of these miRNAs in the antitumor activity of MVs. The prominent role of miR451 and miR31 transfer was indicated by the direct antitumor activity of miR451 and miR31 mimics on HepG2 that resulted in MDR1, MIF, RAB14, and E2F-2 downregulation similar to that of MV-HLSC. Of interest, MDR1, MIF, RAB14, and E2F-2 proteins were overexpressed in HepG2 with respect to normal human hepatocytes.

The antitumor effect was specific of MV-HLSC since the MVs from fibroblast were ineffective. This could depend on a reduced internalization, due to absence of CD29 on MV-fibr or on a reduced expression of antitumor miRNAs. However, when MV-fibr expressing CD29 were used, the enhanced internalization was not associated with an enhanced biological activity. Therefore, it is likely that the different composition of miRNA content between MV-HLSC and MV-fibr was the main cause of the different biological action. On the other hand, the effect of MV-HLSC was not confined to hepatic tumors as they were effective also on lymphoblastoma and glioblastoma tumors.

## CONCLUSIONS

In conclusion, the results of this study suggest that MVs derived from HLSC may inhibit the growth of hepatoma tumors by transferring genetic information that interferes with the deregulated survival and proliferation of these cells. This biological effect could be ascribed to specific miRNAs shuttled by MV-HLSC that are able to modulate signaling pathways differentially activated in tumor cells in respect to normal cells. Therefore, one can speculate that the specificity of biological signal triggered by stem cell-derived MVs depends on the recipient cells and not only on the genetic information transferred by the cell of origin. These results have possible implication on the development of a therapeutic strategy based on MV-mediated delivery of miRNAs [57] present in normal stem cells but not in tumor cells.

## Supplementary material

Supplemental Figure 1. (A) qRT-PCR analysis (left panel) of fibroblasts transfected with pCMV-Sport 6 plasmid carrying the human CD29 (fibr-CD29) full length cDNA or of fibroblasts electroporated in the absence of DNA as control (fibr-CTR). Data were normalized to 1 for fibr-CTR and to GAPDH mRNA. * P < 0.01 vs fibr-CTR. Right panel, Western Blot analysis of CD29 in fibr-CTR, in MVs derived from fibr-CTR, in fibr-CD29 and in MVs derived from fibr-CD29. CD29 transfected fibroblasts released MVs expressing CD29. (B) Nanoparticle tracking analysis (NTA) showing that the RNase treatment (right panel) did not affect MV morphology and size. Left panel shows untreated MV-HLSC. Curve 1 describes the relationship between particle number distribution (left Y axis) and particle size (X axis); curve 2 shows cumulative percentage distribution of particles (percentile in right Y axis) and particle size (X axis). Mean size and particle concentration values were calculated by the NTA software that allow analysis of video images of the particle movement under Brownian motion captured by Nanosight LM10 and calculation of the diffusion coefficient, sphere equivalent, and hydrodynamic radius of particles by using the Strokes-Einstein equation. (C) Measure of selected miRNAs in MVHLSC treated 5U/ml (white bar) of RNase in respect to control MV-HLSC (black bar) expressed as 2-(ΔCt) as described in Methods. Data were normalized to 1 for MV-HLSC and expressed as mean ± SD of 4 different experiments. * P < 0.01 vs MV-HLSC. (D) Measure in MV-HLSC of the levels of different miRNAs relative to the small nucleolar RNA, RNU48 expressed as Log 2-(ΔCt).

Supplemental Figure 2. Evaluation of in vivo tumor proliferation and apoptosis (A) Representative micrographs of PCNA staining of HepG2 tumors. Scale bar, 50 μm. (B) Representative micrographs of Tunel staining. Scale bar, 50 μm.

Supplemental Figure 3. Comparative expression of specific miRNAs and correspondent targets between HepG2 and normal human hepatocytes. (A) Log of relative expression of miR451, miR223, miR31 and miR21 in normal human hepatocytes (Hep; white bar) in respect to HepG2, evaluated by qRT-PCR. (B) qRTPCR analysis of miR451 and miR31 correspondent targets in HepG2 (grey bar) and in Hep (white bar). Data (A and B) were normalized to 18S ribosomal 1 mRNA and to 1 for HepG2 and expressed as mean ± SD of 4 different experiments. * P < 0.05 vs HepG2.
