# Supplementary figures and images for: Human Liver Stem Cell-Derived Microvesicles Inhibit Hepatoma Growth in SCID Mice by Delivering Antitumor MicroRNAs
Source: Stem Cells. 2012 Jun 26;30(9):1985–98. doi: 10.1002/stem.1161 (PMC3468738; doi:10.1002/stem.1161)

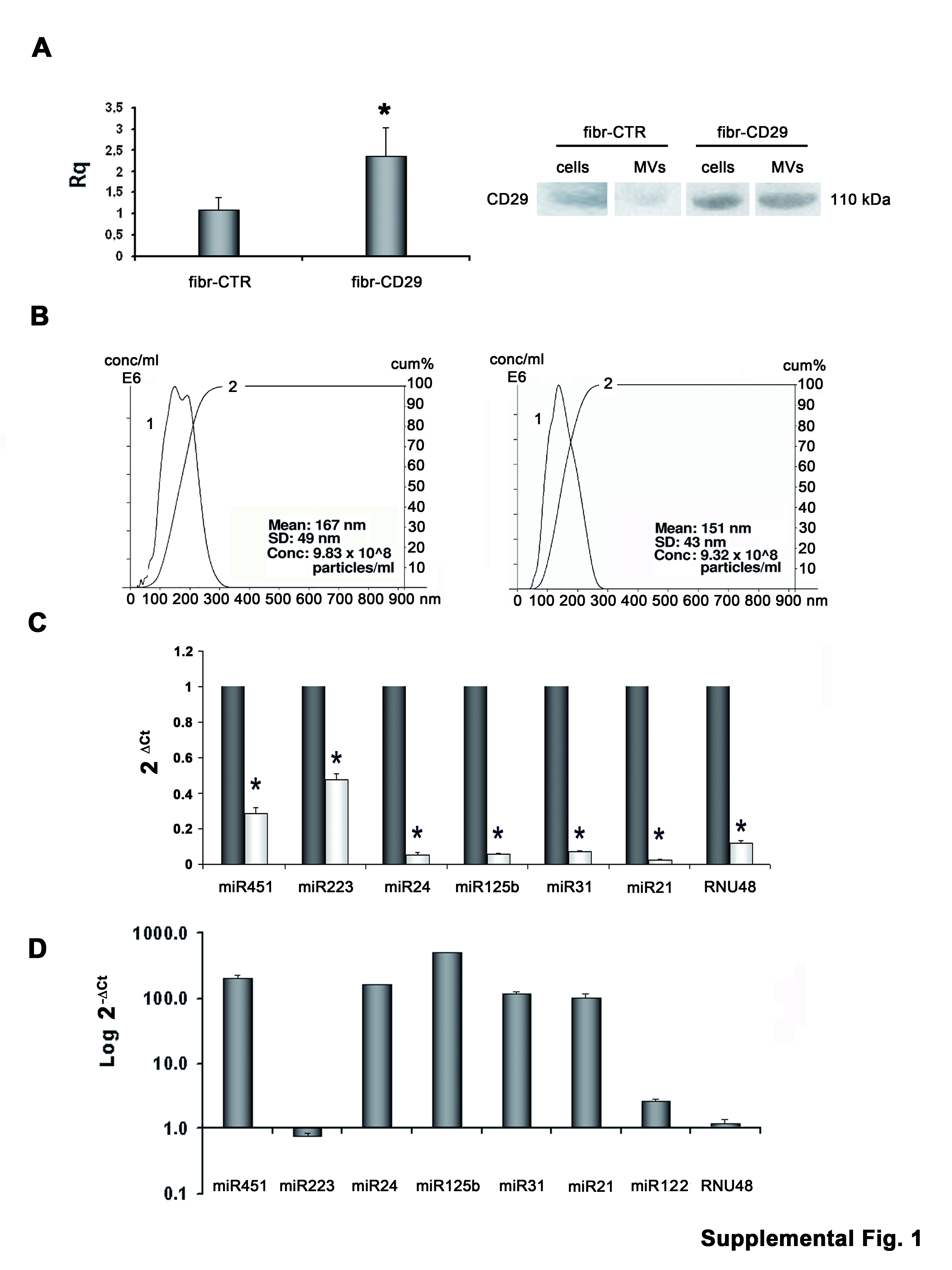

Supplement: Supplementary file 1 [file stem0030-1985-SD1.tif]

**A****HepG2****MV-HLSC**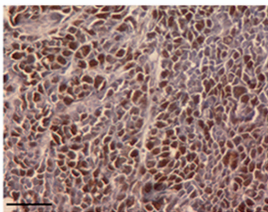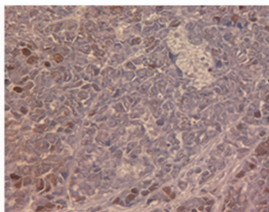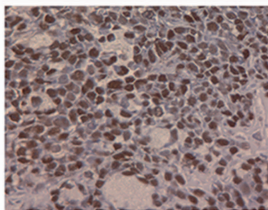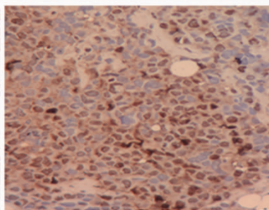**MV-RNase****MV-CD29****B****HepG2****MV-HLSC**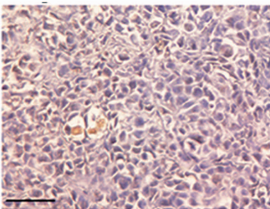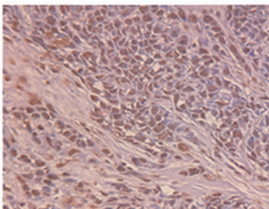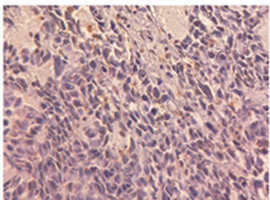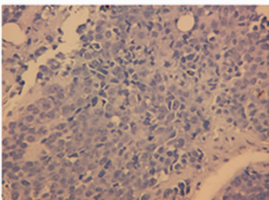**MV-RNase****MV-CD29****Supplemental Fig. 2**

Supplement: Supplementary file 2 [file stem0030-1985-SD2.pdf]

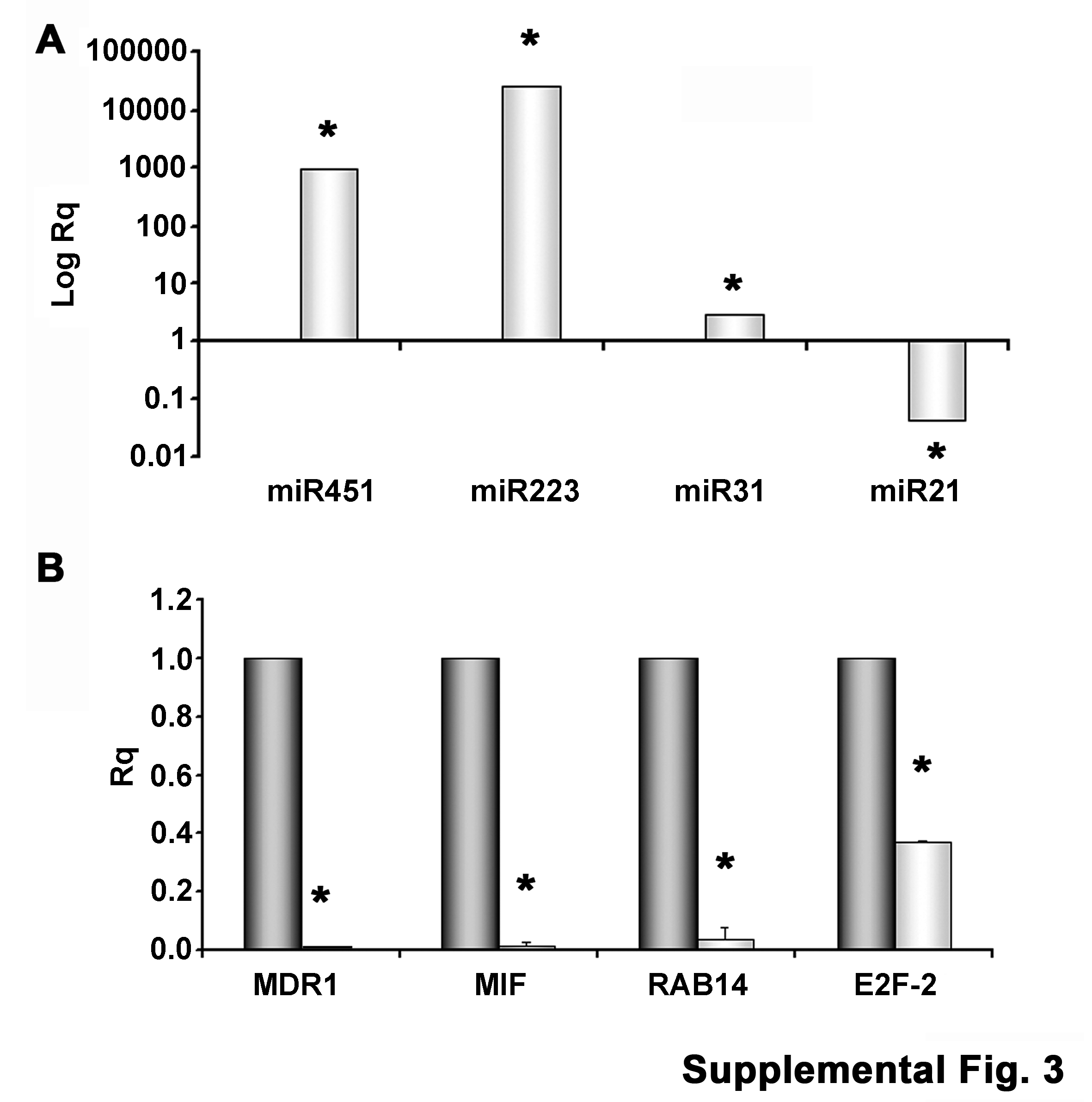

Supplement: Supplementary file 3 [file stem0030-1985-SD3.tif]
