# Supplementary material for: Human Liver Stem Cell-Derived Microvesicles Inhibit Hepatoma Growth in SCID Mice by Delivering Antitumor MicroRNAs
Source: Stem Cells. 2012 Jun 26;30(9):1985–98. doi: 10.1002/stem.1161 (PMC3468738; doi:10.1002/stem.1161)
Supplement: Supplementary file 4 [file stem0030-1985-SD4.pdf]

Supplemental Table 1.

Primers used in qRT-PCR experiments.

Name	Official symbol	Sequences
**Dicer 1, ribonuclease type III**	**DICER1**<br>**(DCR1)**	Forward 5'-ACC CAT ACC TCA GAT TCC ACA CTT-3'<br>Reverse 5'-CTC AAG CAT TTG TAG AGA CAA CAT GA-3'
**Eukaryotic translation initiation factor 2C**	**EIF2C2**<br>**(AGO2)**	Forward 5'- TAT TGA TGT GTC AGC AAC AGC GT -3'<br>Reverse 5'- TTA CCC TTT GGG AAT CTG TCA GAG -3'
**ATP-binding cassette, sub-family B, member 1**	**ABCB1**<br>**(MDR1)**	Forward 5'- CTC AGA CAG GAT GTG AGT TGG TTT -3'<br>Reverse 5'-CCT GGA ACC TAT AGC CCC TTT AA-3'
**Macrophage migration inhibitory factor**	**MIF**	Forward 5′-GCC CGG ACA GGG TCT ACA-3'<br>Reverse 5′-GCG AAG GTG GAG TTG TTC CA-3'
**RAB14, member RAS oncogene family**	**RAB14**	Forward 5′-AGT GCA AAA ACG GGA GAG AAT GT-3'<br>Reverse 5′-ACT CAG CAG CAT TCA GAT CCA AG-3'
**E2F transcription factor 2**	**E2F-2**	Forward 5'-CCG TGC TGT TGG CAA CTT TAA-3'<br>Reverse 5'-TGC AGG TTG TCC TCA GTC CTG-3'
**Integrin, beta 1**	**ITGB1**<br>**(CD29)**	Forward 5'-TCAGAATTGGATTTGGCTCATTT-3'<br>Reverse 5'-TGGTGCAGTTCTGTTCACTTGTG-3'
**RNA, 18S ribosomal 1**	**RN18S1**	Forward 5′-CCT GCC AGT AGC ATA TGC TTG TC-3‘<br>Reverse 5′-AGC CAT TCG CAG TTT CAC TGT A-3‘
**Glyceraldehyde-3-phosphate dehydrogenase**	**GAPDH**	Forward 5′- TGG AAG GAC TCA TGA CCA CAG T -3‘<br>Reverse 5′- CAT CAC GCC ACA GTT TCC C-3‘
